# Sesamin Induces Human Leukemic Cell Apoptosis via Mitochondrial and Endoplasmic Reticulum Stress Pathways

**DOI:** 10.4021/wjon2010.03.195w

**Published:** 2010-04-30

**Authors:** Ratana Banjerdpongchai, Siriporn Yingyurn, Prachya Kongtawelert

**Affiliations:** a Excellent Center for Tissue Engineering, Department of Biochemistry, Faculty of Medicine, Chiang Mai University, Chiang Mai 50200, Thailand

**Keywords:** Sesamin, Human leukemic cells, Endoplasmic reticulum stress, Mitochondrial pathway, Apoptosis

## Abstract

**Background:**

Sesamin is a purified compounds extracted from the seeds of *Sesamum orientale* Linn., which contains antioxidant and anticancer activities. The objective of this study was to identify the mechanistic effect of sesamin on human leukemic HL-60, U937 and Molt-4 cell apoptosis.

**Methods:**

The cytotoxicity was performed by 3-(4,5-dimethyl)-2,5-diphenyl tetrazolium bromide (MTT) assay. Reactive oxygen species was measured by employing 2', 7'-dichlorodihydrofluorescein diacetate and flow cytometry. The mitochondrial transmembrane potential was determined by 3,3'-dihexyloxacarbocyanine iodide and flow cytometer. Caspase-3 and -8 activities were detected by using fluorogenic DEVD-AMC and IETD-AMC substrates, respectively. The protein expression of cytochrome c and GADD153, an endoplasmic reticulum (ER) stress protein, was illustrated by immunoblot.

**Results:**

Sesamin was cytotoxic to HL-60 > U937 > Molt-4 > PBMCs and caused the three cell lines to die with the morphology of apoptotic character, i.e., condensed nuclei and apoptotic bodies. It produced reactive oxygen species in all cell lines, with a decrease in mitochondrial transmembrane potential. The caspase-3 activity was increased in sesamin-induced HL-60 cell apoptosis whereas casase-8 activity did not alter. Cytochrome c release was not increased. The expression of GADD153 was increased time dependently, indicating the involvement of ER stress pathway in HL-60 cells.

**Conclusions:**

Sesamin-induced human leukemic cell apoptosis was via oxidative stress, the mitochondrial and ER stress pathways.

## Introduction

Apoptotic cell death differs from necrosis in various aspects, i.e., cytosol shrinkage, small and condensed nuclei, cell membrane blebbing and fragmentation of nuclei and cytosol to become fragmented bodies or apoptotic bodies. Apoptotic cells are engulfed by macrophages and neighboring cells. Apoptosis does not induce inflammatory process to occur, in contrary, necrotic cells induce inflammation. Necrotic cells lose membrane integrity and allow water and co-compounds to go inside the cell, then the cells swell and finally burst. The substances inside the necrotic cells leak to the environment, which lead to inflammation [1].

Mechanism of apoptotic death can be divided into two main pathways, viz., extrinsic and intrinsic pathways [2]. The extrinsic pathway is signaled from Fas ligand or tumor necrosis factor or tumor necrosis-factor related apoptosis-inducing ligands (TRAIL) binding to its receptor, which is Fas (APO-1 or CD95), tumor necrosis factor receptor, or TRAIL receptor, respectively. The binding triggers receptor oligomerization and signals to the cytosolic part of the death receptor called death domain (DD) and to interact with Fas associated death domain (FADD) or TNF receptor associated death domain (TRADD) and activate caspase-8. Activated caspase-8 relays and cleaves caspase-3, -6 and -7 to become active and cut its substrates such as cytoskeletal proteins to change the morphology of cells to be apoptotic cells [3-6].

The apoptosis causing agents in intrinsic pathway are ionizing radiation, reactive oxygen species (ROS), growth factor withdrawal and DNA damage from chemotherapeutic agents. The signaling is through mitochondria, where Bcl-2 family proteins locate on its membrane. These include anti-apoptotic proteins such as Bcl-xL, Bcl-2, and Mcl-1 and proapoptotic proteins, e.g., Bad, Bax, Bak, and Bok/MTD. In homeostasis, anti-apoptotic proteins bind to pro-apoptotic proteins, the cells survive. If the rheostat changes, pro-apoptotic proteins increase and form dimers and channels at mitochondrial membrane, causing the reduction of mitochondrial transmembrane permeability (MTP) and cytochrome c leakage from intermembranous space into cytosol. Cytochrome c forms complex with apoptotic protease activating factor-1 (Apaf-1) and procaspase-9 to become apoptosome, which cleaves and activate procaspase-9 to become active caspase-9 and then cut and activate executioner caspases (i.e., caspase-3, -6 and -7) [7-9].

Endoplasmic reticulum is an organelle in cytosol that regulates protein synthesis, posttranslational modification, and proper protein folding and enabling secretory or transmembrane proteins to function precisely and correctly. Since there are many proteins and chaperones in endoplasmic reticulum (ER) restore proteins in the form ready to function and prevent them from assembling as aggregates. However, in the toxic conditions, e.g., hypoxia, nutrient deprivation, disulfide bond reduction and overexpression of some proteins causing protein misfolding via ER disruption. Hence, proteins do not fold and accumulate to be aggregates in ER. This phenomenon is called ER stress [10-11].

ER stress stimulates self-defense mechanism of cells by (1) regulation of gene transcription in control chaperones and enzymes in ER to increase functions of protein folding, (2) decrease protein synthesis in translation level to eliminate defective folded proteins and (3) degradation of abnormal folding protein from ER [12]. In mammals there are three ER transmembrane proteins, i.e., inositol requiring 1, activating transcription factor 6, and protein kinase-like ER-resident kinase (PERK). These proteins function as receptors of ER stress and finally stimulate transcription factor to induce chaperones such as glucose-regulated protein 78/immunoglobulin heavy chain binding protein (GRP78/Bip) or to phosphorylate eukaryotic initiation factor 2 (eIF2) to inhibit new protein synthesis. These processes are called unfolded protein response (UPR). The preliminary result of UPR stimulation is to protect ER but it limits the other organelle damage and protects living organisms by getting rid of prolonged stress cells. There are many mechanisms to explain the relationship between ER stress and apoptosis [13]. ER stress stimulates caspase-12 and C/EBP homologous protein/growth arrest and DNA damage-inducible gene 153 (CHOP/GADD153), which transduce in cascades specific for ER stress and induces apoptosis [10, 11]. Schroder et al have reported caspase-12 is activated in mice [11]. In human caspase-12 is mutated in several points but human caspase-4 which locates in ER is compatible to caspase-12 in mice and is stimulated in ER stress [14].

Sesamin is a kind of sesame lignans which is purified from seeds of *Sesamum orientale* Linn. Sesamin is the most abundant lignan in sesame seed and found in various medicinal plants [15, 16]. Sesamin enhances hepatic detoxification of chemicals [17], reduces the incidences of chemically induced tumors [18], and protects neuronal cells against oxidative stress [19, 20]. Sesamin exhibits anti-hypertensive [21], anti-inflammatory [15, 22-24], and anti-allergic effect [25].

Yokota et al have reported that sesamin causes cell cycle arrest at G1 phase in human breast cancer MCF7 cells and decreases Rb protein phosphorylation. Furthermore, sesamin reduces cyclin D1 expression, which can be inhibited by proteasome inhibitor. The reduced expression of cyclin D1 by sesamin is found in lung cancer, kidney cancer, kerratinocyte cancer, melanoma and bone cancer cells [26].

(+)-Sesamin affects DNA structure by damaging DNA in mammalian CHO K1 and HTC cells to give the positive result for comet assay (single-cell gel electrophoresis) [27]. The moiety of methylene dioxy in the molecular structure of (-)-sesamin has inhibitory effect on the proliferation of human cancer cells by blocking the enzyme activity of phospholipase C [28].

It has been reported that sesamin from the bark of *Acanthopanax senticosus* (From Japan) has growth inhibitory effect and induce apoptosis in human stomach cancer KATO III cells [29]. Sesamin can induce human lymphoblastic leukemic Molt4B cell apoptosis but the mechanism remains unclear [30].

The aim of this study was to study the apoptotic effects of sesamin from *Sesamum orientale* Linn. in the model of using human leukemic HL-60, U937 and Molt-4 cells and to identify the molecular mechanisms involved.

## Materials and Methods

### Reagents

Sesamin, MTT (3-(4,5-dimethyl)-2,5-diphenyl tetrazolium bromide), histoplaque, propidium iodide (PI) , 3,3'-dihexyloxacarbocyanine iodide (DiOC_6_), and 2',7'-dichlorodihydrofluorescein diacetate (DCFH-DA) were obtained from Sigma-Aldrich (St. Louis, MO, USA). RPMI-1640 medium was purchased from Invitrogen, USA. DEVD-AMC and IETD-AMC was from Biosource, USA. Primary antibodies to cytochrome c were obtained from Abcam, Cambridge, UK. Horseradish peroxidase (HRP) conjugated rabbit antibody was from Pierce, Rockford, IL, USA.

### Cell culture

Human promyelocytic leukemic HL-60, human promonocytic U937 and human lymphoblastic Molt-4 cells were cultured in 10% fetal bovine serum in RPMI-1640 medium supplemented with penicillin G (100 units/ml) and streptomycin (100 ug/ml) at 37°C in a humidified atmosphere containing 5% CO_2_. The preconfluent (growth phase) cells (1 x 10^6^ cells) were treated with sesamin at concentrations of 10 - 400 ug/ml.

Peripheral blood mononuclear cells (PBMCs) were isolated from heparinized blood obtained from adult volunteers by density gradient centrifugation using histoplaque according to standard protocols. After separation, cells were cultured in RPMI-1640 medium supplemented with 10% heat-activated fetal bovine serum, 2 mM glutamine, 100 U/ml penicillin, and 100 ug/ml streptomycin. PBMCs were treated with sesamin at concentrations of 10 - 400 ug/ml.

### Cytotoxicity test

MTT assay was performed as described briefly [30]. Following sesamin treatment, cell viability was assessed by MTT assay. This method is based on the ability of viable cells to reduce MTT (3-(4,5-dimethyl)-2,5-diphenyl tetrazolium bromide) and form a blue formazan product. MTT solution (sterile stock solution of 5 mg/ml) was added to the incubation medium in the wells at a final concentration of 100 ug/ml and incubated for 4 h at 37 °C in a humidified 5% CO_2_ atmosphere. The medium was then removed and plate was shaken with DMSO for 30 min. The optical density of each well was measured at 540 nm with reference wavelength of 630 nm using microtiter plate reader (Biotek, USA). Number of viable cells was calculated from untreated cells, and the data were expressed as percentage of cell viability.

### Fluorescence microscopy

Treated cells were cytospun on glass slides. After air drying, cells were fixed with absolute methanol for 10 min at -20 °C, washed twice with PBS and air-dried. Propidium iodide (200 ug/ml) was applied to the fixed cells for 10 min at room temperature. After washing with PBS and drying, the slides were mounted with 90% glycerol and examined under fluorescence microscope (Olympus, Japan).

### Determination of mitochondrial transmembrane potential and ROS production

For measurement of mitochondrial membrane potential and intracellular ROS, either 40 nM of 3,3'-dihexyloxacarbocyanine iodide (for mitochondrial membrane potential) or 5 mM of 2',7'-dichlorodihydrofluorescein diacetate (for ROS detection) was added for 15 min at 37 °C and the cells are then subjected to a FACScan equipped with a 488 nm argon laser using CellQuest software (Becton-Dickinson, USA).

### Assays of caspase-3 and -8 activity

Cleavage of the fluorogenic peptide substrates DEVD-AMC and IETD-AMC (indicative of caspase-3-like and caspase-8 like enzyme activity), respectively, were estimated. Cell lysate (1 x 10^6^ cells) and substrate (50 uM) were combined in a standard reaction buffer and added to a 96-well plate. Enzyme-catalyzed release of AMC was measured by a fluorescence plate reader (Bio-tek, USA) using 355 nm excitation and 460 nm emission wavelengths.

### Western blot analysis

To separate the cytosolic-rich fraction, cells treated with sesamin were harvested and washed once in ice cold PBS and incubated at 4°C for 10 min with ice-cold cell lysis buffer (250 mM Sucrose, 70 mM KCl, 0.25% Triton X-100, 100 µM PMSF, 1 mM DTT in PBS with protease inhibitors). The cell suspension was centrifuged at 20,000 *g* for 20 min. The supernatant was collected and kept as the cytosolic-rich fraction. The protein concentration of the lysate was determined by the Bradford method, using BSA as standard. Cellular protein (50 µg protein) were separated by electrophoresis on 17% SDS-PAGE and transferred onto nitrocellulose membranes. After blocking in 5% non-fat milk in TBS containing 0.2% Tween-20, blots were incubated with mouse monoclonal antibody to cytochrome c (1:1,000) and GADD153 (1:200). For detection the appropriate horseradish peroxidase (HRP) conjugated rabbit antibodies were used at 1:20,000. Protein bands were visualized with Super Signal West Pico Chemiluminecent Substrate on an X-ray film.

### Statistical analysis

Results are expressed as mean ± SEM. (standard error of the mean). Statistical differences between controls and treated groups were determined by the one-way ANOVA (Kruskal Wallis analysis) at limit of p < 0.05 in triplicate of three independent experiments.

## Results

### Cytotoxicity test

**Figure 1:**
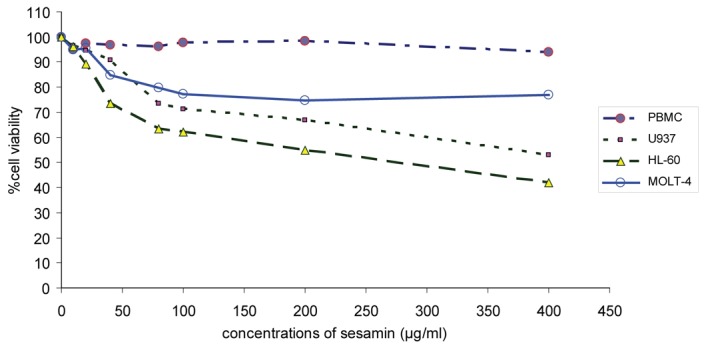
The effect of sesamin on cell cytotoxicity on human leukemic cell lines. Cell viability of human leukemic HL-60, U937 and Molt-4 cells were compared to PBMCs treated with sesamin at various concentrations by using MTT assay.

Sesamin was toxic to HL-60 > U937 > Molt-4 cells by using MTT assay as shown in Figure 1. It did not affect the viability of PBMCs. The IC_50_ value of sesamin for HL-60 cells was 265 ug/ml, whereas it did not reach IC_50_ for U937 and Molt-4 cells at the concentrations tested (> 400 ug/ml). The maximal concentration used was 400 ug/ml due to the limitation of sesamin solubility in DMSO.

### Cell morphology

**Figure 2:**
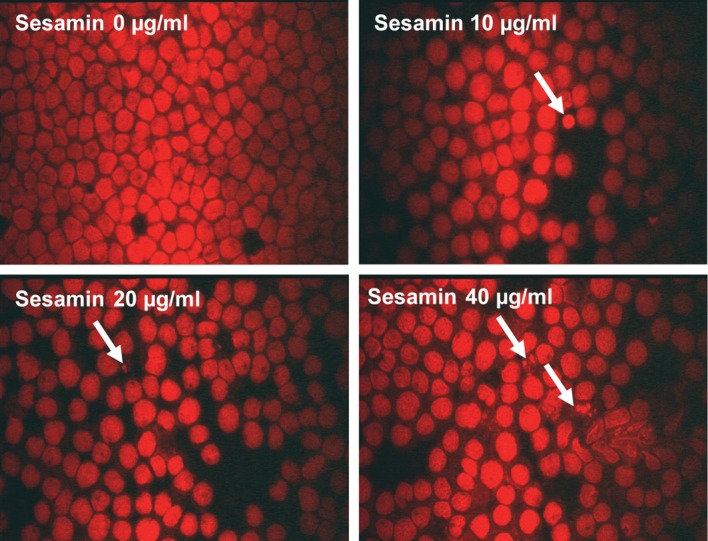
Cell morphology of U937 cells after treatment with sesamin. The cells were treated with sesamin at indicated concentrations for 24 h and stained with propidium iodide and examined under fluorescence microscope. Condensed nuclei and apoptotic bodies were marked with arrows. Magnificaiton x 400.

For further experiments, the concentrations of sesamin used were 10, 20 and 40 ug/ml, which gave the cell viability more than 70%. After treatment of human leukemic HL-60, U937 and Molt-4 cells with sesamin and the cell morphology was examined under fluorescence microscope after staining the nuclei with propidium iodide. It was found that the nuclei condensed and became fragmented to be apoptotic bodies as shown in Figure 2 of U937 cells (for HL-60 and Molt-4 apoptotic cell morphologies were alike), where as those of PBMCs were not determined because (1) sesamin was not cytotoxic to them and (2) the heterogeneity of cells in the cell population, i.e., neutrophils, eosinophils, basophils, monocytes, and lymphocytes.

### Determination of mitochondrial transmembrane potential (MTP)

**Figure 3:**
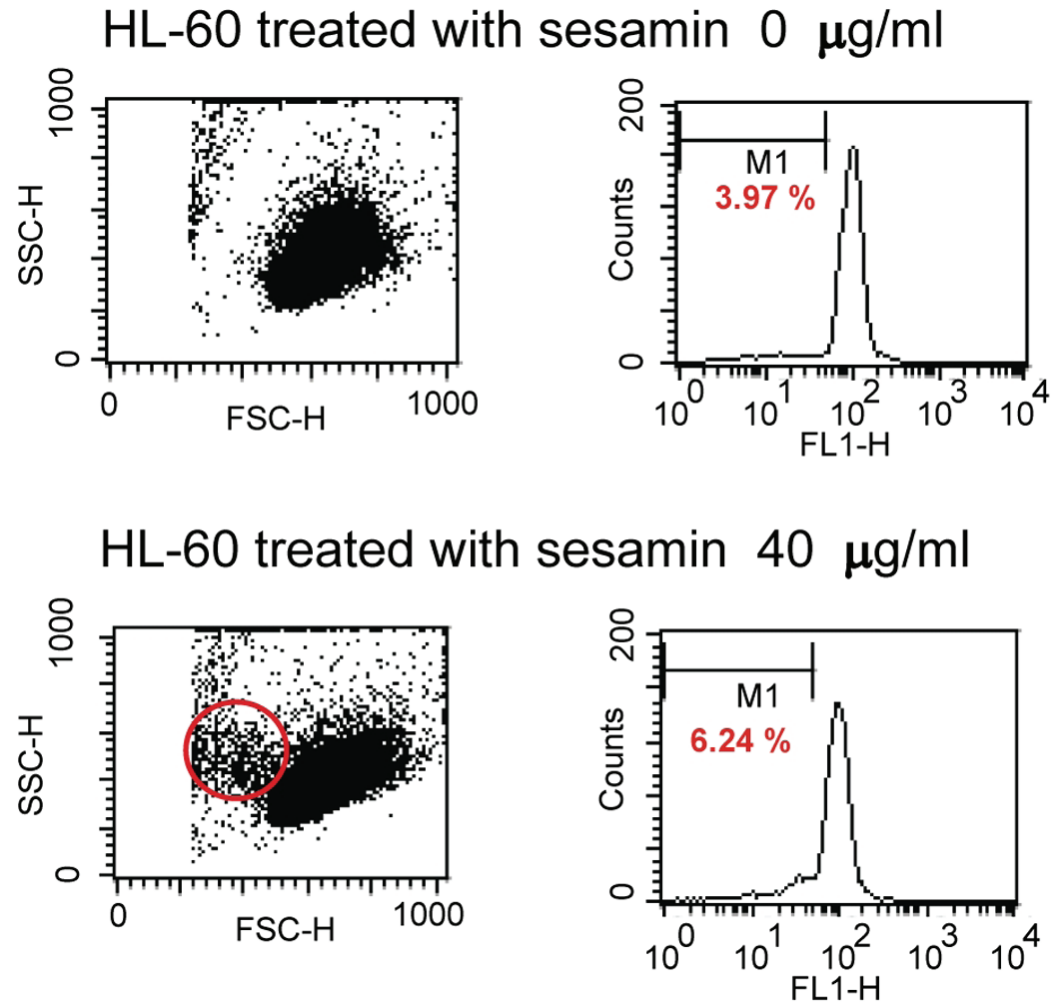
Dot plots and histograms of HL-60 cells treated with sesamin. The cells were stained with 40 nM of 3,3'-dihexyloxacarbocyanine iodide and detected by flow cytometry. M1 area represents percentage of cells with decreased MTP. Circle area is cells with smaller size which are apoptotic cells.

**Figure 4:**
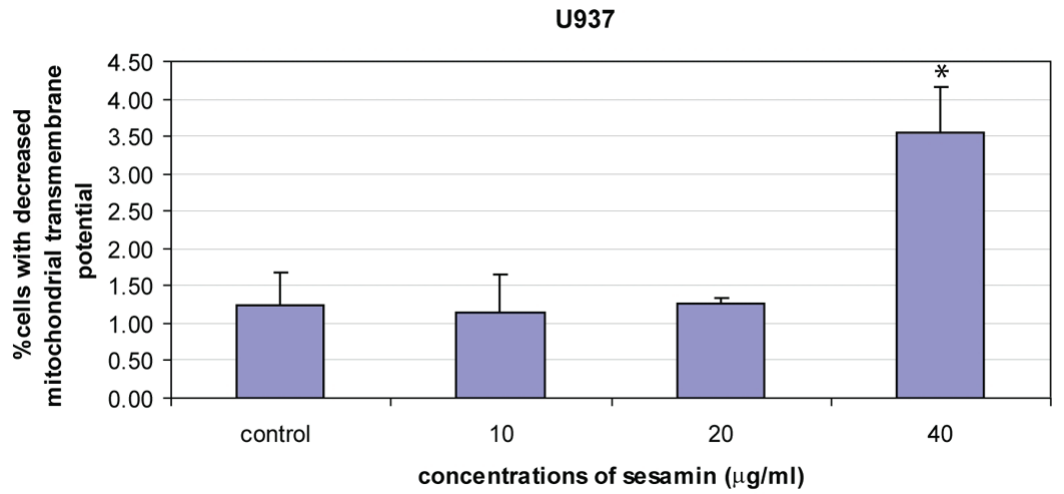
The reduction of mitochondrial transmembrane potential of U937 cells treated with sesamin for 4 h. Percent cells with reduced MTP were increased in a dose response manner. The data were means ± S.E.M. of three independent experiments performed in duplicate. p < 0.05, (*) significant difference from control.

When treated HL-60 cells with sesamin for 4 h and stained with DiOC_6_, cells were fragmented as shown in circle area in Figure 3 and MPT reduced as shown with percent cells with decreased fluorescence intensity (M1 area). It was found that percent human leukemic U937 cells with decreased MTP was increased when treated with sesamin as shown in Figure 4 whereas that of Molt-4 did not change and HL-60 cells was the same as U937 cells (data not shown).

**Figure 5:**
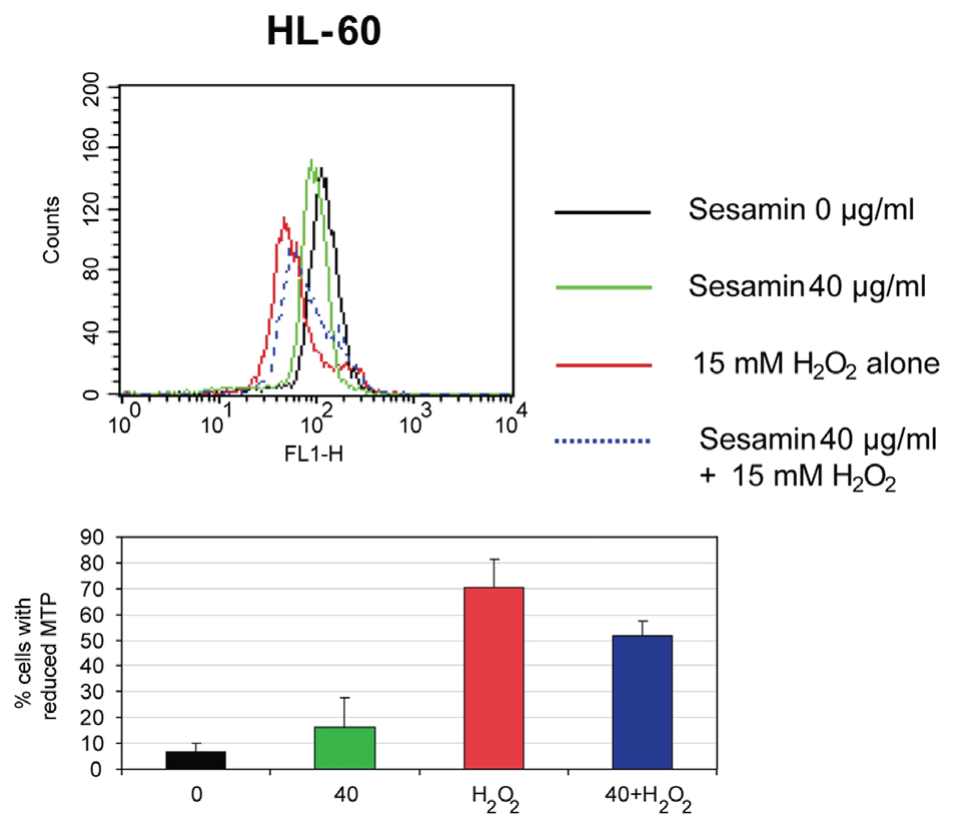
The reduction of MTP of HL-60 cells when treated with sesamin for 1 h. The histograms of cells when treated with H_2_O_2_, sesamin at 40 ug/ml and combined treatment (upper panel) and percentages of cells with decreased MTP in each condition (lower panel) are shown.

When the cells were treated with sesamin (40 ug/ml) for 1 h compared to hydrogen peroxide (H_2_O_2_) treatment at 15 mM for 1 h and combined treatment, there was a reduction of MTP in sesamin, in H_2_O_2_ and combined treatment (Fig. 5).

### Determination of ROS production

**Figure 6:**
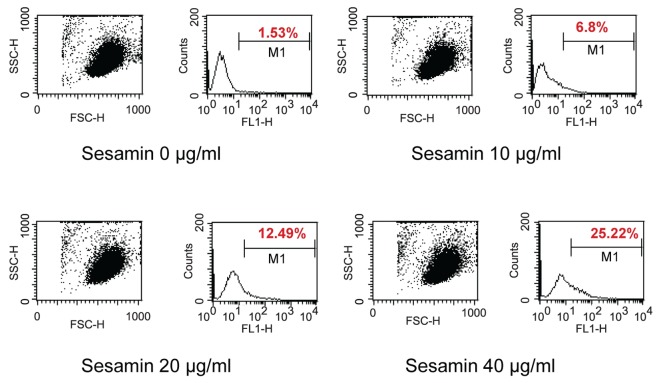
The effect of sesamin treatment on ROS production in human leukemic cells. Dot plot and histogram of HL-60 cells treated with sesamin at indicated concentrations for 4 h. The cells that stained with DCFH-DA were cells producing ROS and existed under area M1. It was in a dose response manner of increasing in production of free radicals.

**Figure 7:**
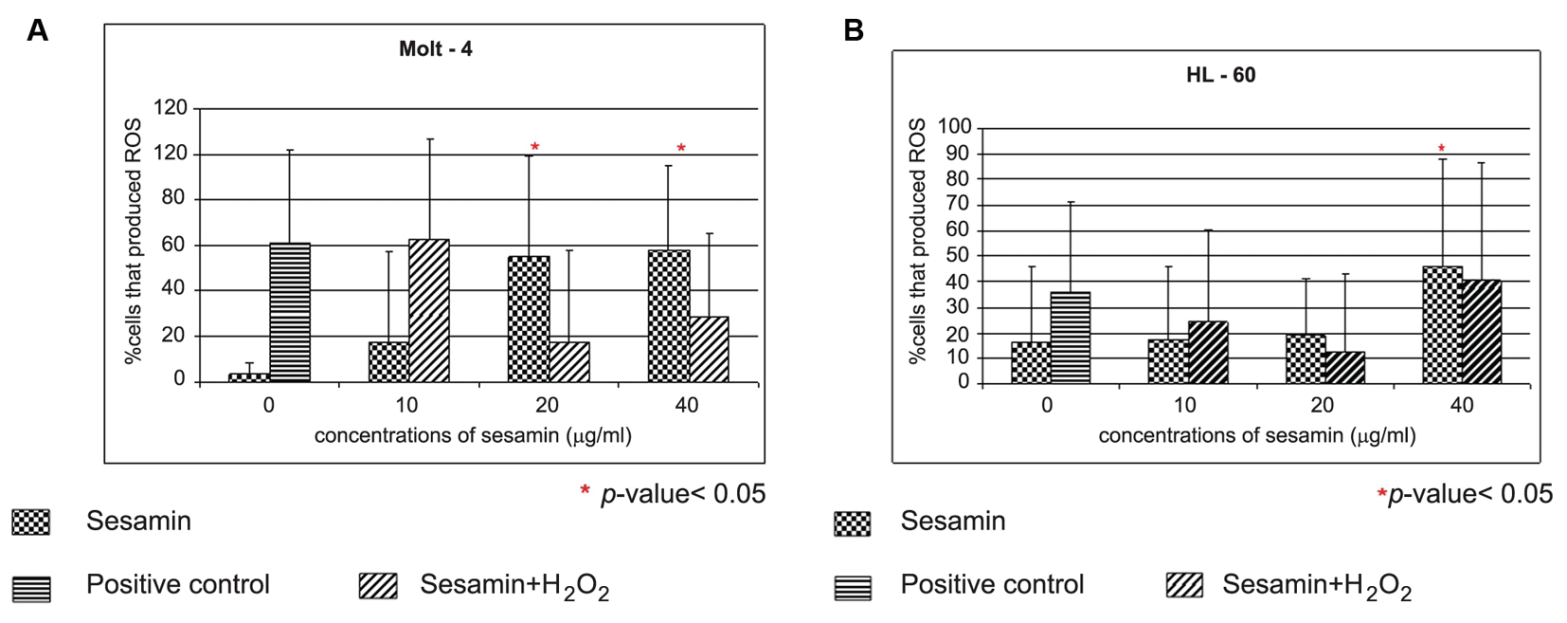
The effect of sesamin on ROS production in Molt-4 cells and HL-60. The Molt-4 (A) and HL-60 (B) cells were treated with sesamin at indicated concentrations for 4 h and in combined treatment with H_2_O_2_. Positive control was the treatment with 15 mM H_2_O_2_ alone. The data were performed in three independent experiments and represented as mean ± S.E.M. p < 0.05 (*) is considered as significant difference compared to control.

Sesamin-treated HL-60 cells showed a dose response in ROS production as shown in Figure 6. The Molt-4 cells treated with sesamin, the cells produced ROS in a dose response manner (p < 0.05) and sesamin acted an antioxidant at doses of 20 and 40 ug/ml in combined treatment with H_2_O_2_. However, at doses of 10 ug/ml, it enhanced the effect of H_2_O_2_, i.e., sesamin acted as pro-oxidant at this concentration in combined treatment in Molt-4 cells (Fig. 7A). In combined treatment with H_2_O_2_, it was found that it reduced the effect of H_2_O_2_ alone at the sesamin concentrations of 20 and 40 ug/ml as shown in Figure 7B. At 10 ug/ml sesamin acted as prooxidant to enhance the effect of H_2_O_2_.

### Assay of Caspase-3 and caspase-8 activity

**Figure 8:**
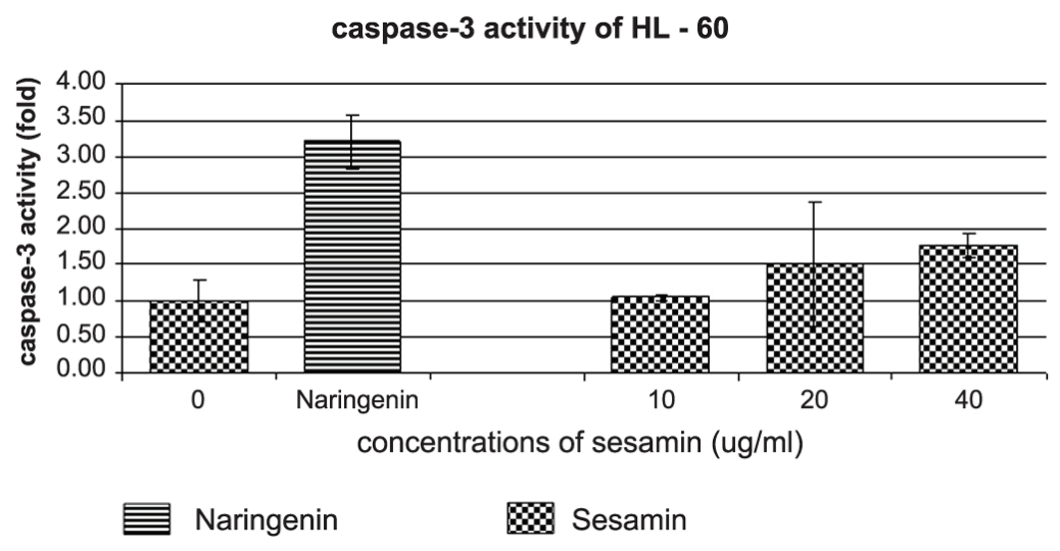
The effect of sesamin on caspase-3 activity of HL-60 cells. An increase of caspase-3 activity of HL-60 cells treated with sesamin at indicated concentrations are determined by DEVD-AMC substrate. Naringenin at 250 ug/ml was used as positive control for caspase-3 activity induction in HL-60 cells.

The caspase-3 and -8 activities were determined by using DEVD-AMC and IETD-AMC substrate kits, respectively. Sesamin-treated HL-60 showed a dose response pattern in caspase-3 activity (Fig. 8), whereas caspase-3 activity did not change in U937 and Molt-4 cells. Meanwhile the activity of caspase-8 in three cell lines did not change compared to negative control (data not shown).

### Cytochrome c release from mitochondria to cytosol

It was found that the expression of cycochrome c of 4 h sesamin treatment at indicated concentrations did not alter compared to the control and the expression of cytochrome c at various times was not changed as well compared to control (data not shown).

### The expression of GADD153

**Figure 9:**
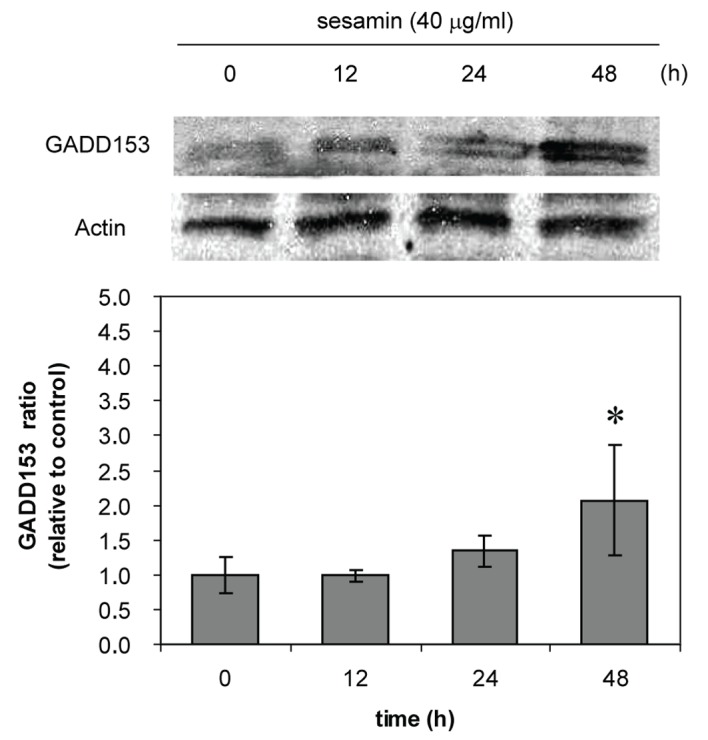
GADD153 expression of sesamin-treated HL-60 cells. The cells were treated with sesamin 40 ug/ml at indicated times. The upper panel shows the bands from immunoblot of GADD153 compared to actin as a constitutive protein. The lower panel represents the data as mean ± S.E.M. p < 0.05 (*) considered significant difference compared to control.

In further experiment of determining the GADD153 expression, HL-60 cells were chosen to be studied in more details for the mechanism of cell death because their caspase-3 activity increased compared to U937 and Molt-4 cells (of which the activities did not alter). The expression of GADD153 at 4 h sesamin treatment at various concentrations did not differ from control (without treatment) but when treated with sesamin 40 ug/ml at various times, the expression increased time dependently (Figure 9). The increase of expression of GADD153 indicates the ER stress pathway of apoptosis.

## Discussion

Sesamin was cytotoxic to the human leukemic HL-60, U937 and Molt-4 cells whereas it did not affect the viability of PBMCs. The transformed or leukemic cells were susceptible to sesamin in the order as follows: HL-60 > U937 > Molt-4 cells. The underlying mechanism remains to be further elucidated. In low dose of sesamin at 10, 20 and 40 ug/ml, it induced apoptotic changes in cell morphology when stained with propidium iodide and examined under fluorescence, viz. condensed nuclei and fragmented bodies (apoptotic bodies).

The mitochondrial membrane potential was reduced dose-dependently in the sesamin-treated U937 cells compared to HL-60 and Molt-4 cells which was little changed and not in a dose response manner at 4 h sesamin treatment. However, at 1 h treatment, the cells showed the reduction of MTP significantly in the three cell lines. In the latter condition, sesamin acted as an antioxidant in the co-treatment with H_2_O_2_ condition compared to the condition treated with H_2_O_2_ alone.

HL-60 and Molt-4 cells treated with sesamin produced ROS significantly. However, sesamin or sesame lignans are antioxidants but shows pro-oxidant effects when their concentration up to 100 µM [19, 20]. Sesamin suppresses lipid peroxidation of erythrocytes [31, 32] and prevent hypoxic or H_2_O_2_ stressed death of neuronal PC12 cells [20]. The antioxidant or pro-oxidant effect of sesamin seemed to depend on cell types and doses of sesamin.

Caspase enzymes are composes of various kinds and could be used to identify for the pathways of apoptosis whether it involves extrinsic or intrinsic pathways. Caspase-3 is an executioner caspase that cleaves cytoskeletal proteins and causes the morphological changes of apoptotic cells. It was found that caspase-3 activity increased in sesamin-treated HL-60 cells. For caspase-8 which involves the extrinsic pathway, the caspase-8 activity did not alter in the three leukemic cell lines, thus excluding the death receptor involvement of sesamin-induced leukemic apoptosis.

The MTP was reduced indicating the involvement of mitochondria. To confirm this, cytochrome c release into cytosol was determined by immunoblotting. It was found that the expression of cytosolic cytochrome c did not significantly change at 4 h of treatment at concentrations 10, 20 and 40 ug/ml and not in a time dependent manner (data not shown).

It was reported that curcumin induces pro-apoptotic endoplasmic reticulum stress in human leukemic HL-60 cells [33]. To demonstrate the ER stress in sesamin-treated HL-60 cells, GADD153 expression was determined. There was an increase in GADD153 protein expression at 12, 24 and 48 h of 40 ug/ml sesamin-treated HL-60, indicating ER stress involvement.

Sesamin-treated human HL-60 and Molt-4 cells produced ROS but in condition of combined treatment with H_2_O_2_, sesamin has dual effect, as an antioxidant or a prooxidant depending on the concentrations of sesamin and types of leukemic cells. Taken together, the sesamin-induced apoptotic death pathways involved mitochondrial transmembrane potential reduction, caspase-3 activation and increased expression of GADD153, which was an ER stress protein.

In conclusion, sesamin induced human leukemic cell apoptosis via ER stress, oxidative stress and mitochondrial pathways. The therapeutic application with conventional therapy requires further experiments in *in vivo* model.
